# Meta‐QTL s and haplotypes for efficient zinc biofortification of rice

**DOI:** 10.1002/tpg2.20315

**Published:** 2023-03-10

**Authors:** Gaurav Joshi, Yan Paing Soe, Alvin Palanog, Tapas Kumer Hore, Chau Thanh Nha, Mark Ian Calayugan, Mary Ann Inabangan‐Asilo, Amery Amparado, Indra Deo Pandey, Pompe C. Sta Cruz, Jose E. Hernandez, B. P. Mallikarjuna Swamy

**Affiliations:** ^1^ Rice Genetic Design and Validation Unit International Rice Research Institute Los Baños Philippines; ^2^ Govind Ballabh Pant University of Agriculture and Technology Pantnagar Uttarakhand India; ^3^ Department of Agriculture Nay Pyi Taw Myanmar; ^4^ Can Tho University (CTU) Can Tho Vietnam; ^5^ Philippines Rice Research Institute Muñoz Nueva Ecija Philippines; ^6^ University of the Philippines Los Banos Laguna Philippines

## Abstract

Biofortification of rice with improved grain zinc (Zn) content is the most sustainable and cost‐effective approach to address Zn malnutrition in Asia. Genomics‐assisted breeding using precise and consistent Zn quantitative trait loci (QTLs), genes, and haplotypes can fast‐track the development of Zn biofortified rice varieties. We conducted the meta‐analysis of 155 Zn QTLs reported from 26 different studies. Results revealed 57 meta‐QTLs with a significant reduction of 63.2% and 80% in the number and confidence interval of the Zn QTLs, respectively. Meta‐quantitative trait loci (MQTLs) regions were found to be enriched with diverse metal homeostasis genes; at least 11 MQTLs were colocated with 20 known major genes involved in the production of root exudates, metal uptake, transport, partitioning, and loading into grains in rice. These genes were differentially expressed in vegetative and reproductive tissues, and a complex web of interactions were observed among them. We identified superior haplotypes and their combinations for nine candidate genes (CGs), and the frequency and allelic effects of superior haplotypes varied in different subgroups. The precise MQTLs with high phenotypic variance, CGs, and superior haplotypes identified in our study are useful for an efficient Zn biofortification of rice and to ensure Zn as an essential component of all the future rice varieties through mainstreaming of Zn breeding.

## Core Ideas

- Meta‐analyses of 155 Zn QTLs identified 57 MQTLs with reduced confidence intervals.
- Major metal homeostasis genes colocated with MQTLs were prioritized.
- In silico expression and co‐expression analyses revealed the complex network of genes involved in metal homeostasis.
- Identified superior haplotypes are useful for efficient rice Zn biofortification.

**Abbreviations**
3K‐RGP: 3K rice genome panel
AIC: Akaike information criterion
BLUEs: best linear unbiased estimations
CG: candidate genes
CI: confidence interval
GAB: genomics‐assisted breeding
GO: gene ontology
GWAS: genome‐wide association studies
HSD: honestly significant difference
LOD: logarithm of odds
MAS: marker‐assisted selection
MQTLs: meta‐quantitative trait loci
MR: mutual rank
QTLs: quantitative trait loci

## 1 INTRODUCTION

Zinc (Zn) is one of the most important mineral elements required for the successful completion of life cycle in both plants and animals (Yashona et al., 2018). It provides structural and functional stabilities to hundreds of enzymes, and proteins thereby play a vital role in cell division, immune function, cognitive development, sensory function, reproductive health, and neurobehavioral development in human beings (Cakmak & Hoffland, 2012; Gibson, 2012; Stein, 2010). Therefore, an adequate daily intake of Zn is recommended for the proper growth, development, and to maintain health. The prolonged Zn deficiency leads to stunting, diarrhea, vulnerability to diseases, poor cognitive development, infant and maternal mortalities, and so on (Brooks et al., 2004; Calayugan et al., 2021; Sazawal et al., 2007; Tielsch et al., 2007; Young et al., 2014). These health issues are widespread globally but highly visible in the populations that are entirely dependent on staples for their nutritional needs (Hotz & Brown, 2004; Stein, 2010; Cakmak & Hoffland, 2012; Gibson, 2012). Zn malnutrition affects over two billion people worldwide; especially children, women, and elderly are highly vulnerable. Thus, addressing malnutrition has been accorded a major global health priority (UN General Assembly, 2015).

Biofortification of food crops with enhanced nutritional value is the most cost‐effective and a sustainable food‐based solution to combat malnutrition (Bouis et al., 2011; HarvestPlus, 2021; Siwela et al., 2020; White & Broadley, 2009). As a result, several biofortified crop varieties with higher Zn, Fe, and vitamin A contents have been commercialized, and subsequent bio‐efficacy studies using biofortified crops were found to be effective in improving the nutritional status (De Moura et al., 2014; Sazawal et al., 2018). In rice, several Zn biofortified varieties with an additional 8–10 ppm of grain Zn content have been released for commercial cultivation in the target countries of Asia, and efforts are being made to deploy them widely (Calayugan et al., 2021; Inabangan‐Asilo et al., 2019). However, there is an urgent need to expedite the development of Zn biofortified rice and also to mainstream the grain Zn as an essential component of the rice breeding product profiles, so that all the future rice varieties will have enhanced levels of grain Zn content. A better knowledge of the molecular basis of grain Zn accumulation, factors that influence the stable performance of high Zn lines, and combining a suite of agronomic and other desirable traits along with high grain Zn content are essential for the success of rice Zn biofortification program. Efforts are being made to mainstream Zn in rice breeding by using advanced breeding techniques, such as rapid generation advance, speed breeding, recurrent selection, and multi‐trait genomic selection (Calayugan et al., 2021; CGIAR, 2018).

**Core Ideas**

- Meta‐analyses of 155 Zn QTLs identified 57 MQTLs with reduced confidence intervals.
- Major metal homeostasis genes colocated with MQTLs were prioritized.
- In silico expression and co‐expression analyses revealed the complex network of genes involved in metal homeostasis.
- Identified superior haplotypes are useful for efficient rice Zn biofortification.

The enormous wealth of genetic resources and molecular information available for rice has enabled the genomic dissection of complex traits, including grain Zn and Fe contents, to identify major quantitative trait loci (QTLs) or genes (Jiang et al., 2012; Swamy et al., 2016; Yang et al., 2013). Although several grain Zn QTLs have already been identified, very few of them have been successfully used in the breeding programs, mainly because of their small effect size, unavailability of diagnostic markers, large confidence intervals (CIs), and many of them have not been either validated or fine mapped (Gong et al., 2018; Mackill, 2006). In addition, QTL identification is highly influenced by the choice of parents, markers, size and type of population, and environmental factors leading to less accuracy or precision of QTLs location or effects, which obstructs their transferability across different breeding programs (Bradshaw et al., 1998, 1995; Garrin et al., 2021; Li et al., 2003; Yu et al., 2011). Thus, an identification of genetically and environmentally stable major effect Zn QTLs and their use in genomics‐assisted breeding (GAB) is necessary to fast‐track the development of Zn biofortified rice varieties.

Meta‐QTL (MQTL) analysis consolidates QTLs mapped for a trait in multiple independent genetic backgrounds and environments to provide precise, stable, and narrow genomic regions for a cluster of QTLs (Arcade et al., 2004; Goffinet & Gerber, 2000). Recently, many studies have reported MQTLs for grain yield, biotic and abiotic stress tolerances, grain quality, and nutrition‐related traits in different crops (Gudi et al., 2022; Guo et al., 2018; Saini et al., 2022; Sheoran et al., 2022; Soriano et al., 2021). MQTLs with improved genetic resolution have been identified for grain Zn and Fe contents in rice, wheat, maize, and common beans (Izquierdo et al., 2018; Jin et al., 2015, 2013; Raza et al., 2019; Shariatipour et al., 2021; Singh et al., 2022). All these studies have clearly shown that MQTL analysis is a powerful tool to identify stable QTLs with narrow CI. Particularly in rice, there are already 2 studies reporting 22 and 46 MQTLs for grain Zn content, and 7 of these MQTLs were common; there was a 75% reduction in the total number of QTLs, and also, their CIs were significantly reduced (Jin et al., 2015; Raza et al., 2019). But recently, single‐nucleotide polymorphisms (SNPs) have been widely used in QTL mapping and genome‐wide association studies (GWAS) for grain Fe and Zn contents in rice. Thus, there is a need for an updated meta‐analysis by consolidating Zn QTLs identified using high‐density maps.

Major effect MQTLs are more useful for GAB and to mine candidate genes (CGs) for designing functional markers and for their functional characterization (Collard & Mackill, 2008; Islam et al., 2019). With the availability of multi‐omics platforms, now it is easier to understand the functions of CGs, to assess their diversity, and identify superior haplotypes in the germplasm panels such as 3K‐RGP (3K rice genome panel) (Abbai et al., 2019; Bevan et al., 2017; Li et al., 2014; Sato et al., 2011; Varshney et al., 2018).

Therefore, the major objectives of our study were to conduct the review of literature to compile QTLs identified for grain Zn content, to identify consensus and precise Zn‐related QTL regions through MQTL analysis, to mine CGs associated with Zn metabolism and understand their expression pattern, co‐expression network, and to identify superior haplotypes of selected CGs. The results from this study will be useful for the efficient Zn biofortification of rice through GAB.

## 2 MATERIALS AND METHODS

### 2.1 Literature search to compile Zn QTL information

We carried out Google Scholar search to compile QTLs identified for grain Zn in rice; preferably those identified using simple sequence repeats (SSRs) and SNP markers. We retrieved Zn QTL information from 2007 to 2020 using various keywords, such as QTL mapping, association mapping, Zn, minerals, and rice. The unpublished Zn QTL results from MS and Ph.D. thesis conducted at the International Rice Research Institute (IRRI) were also used for the analysis. In all 26 QTL mapping studies were utilized for the analyses. The details of the parental lines used in mapping population development, population size, number and type of markers used, range of Zn content in the population, and number of QTLs identified are presented in Table 1. The QTL information compiled are QTL names, linkage group, peak position, the logarithm of odds (LOD), phenotypic variance explained (*R* ^2^), and CI. Wherever peak QTL position information was lacking, the mid‐point between the two flanking markers was used. Similarly, a LOD threshold of 3 was used for the missing LODs. The following formulas were used to estimate the missing CIs of QTLs:

(I) $$\mathrm{CI}\;=\frac{163}{\left(N\times R^{2}\right)}\;$$

(II) $$\mathrm{CI}\;=\frac{530}{\left(N\times R^{2}\right)}\;\;$$

(III) $$R^{2}=\;1-10^{\left(-\frac{2LOD}{N}\right)}$$

where *N* represents the population size, and *R* ^2^ is the phenotypic variance percentage explained by individual QTL. Equation (I) was used to estimate the CI of recombinant inbred lines, whereas Equation (II) was used for backcross‐derived populations and F_2_ populations (Darvasi & Soller, 1997; Weller & Soller, 2004). Equation (III) was used to calculate missing *R* ^2^ (Nagelkerke, 1991). The CI of the QTLs from the GWAS was considered 0.5 Mb each upstream and downstream from the position of maker trait associations. After gathering all the information, a separate QTL input file was prepared for each chromosome.

**TABLE 1:** Summary of quantitative trait loci (QTLs) studies used in the QTL meta‐analysis for grain Zn in rice.

Population	PT^a^	PS^b^	NM^c^	MT^d^	Zn (ppm)	QTLs	*R* ^2^ (%)^e^	TR^f^	PM^g^	Reference
IR64 × Azucena	DH	129	582	RFLP, SSR	–	2	12.8–15	BR	ICP–OES	Stangoulis et al. (2007)
Teqing × *Oryza rufipogon* Griff.	BILs	85	179	SSR	13.3–60.1	3	5–19	BR	ICAP	Garcia‐Oliveira et al. (2009)
Madhukar × Swarna	RILs	168	519	SSR	0.4–104	6	29–35	BR	AAS	Anuradha et al. (2012)
Chunjiang 06 × TN1	DH	120	178	SSR	13.03–42.16	6	10.8–15.4	BR	ICP–OES	Du et al. (2013)
Jalmagna × Swarna	RILs	126	84	SSR	14–31	17	0.01–6	BR	AAS	Kiranmayi et al. (2014)
PAU201 × Palman579	F_2_	50	100	SSR	4.40–157.40	3	4.7–19.1	BR	AAS	Kumar et al. (2014)
Germplasm	Panel	378	143	SSR	16.1–43.14	5	2.3–3.9	BR	ICP–MS	Huang et al. (2015)
IR75862 × Ce258	BILs	201	129	SSR	–	5	2–24.8	MR	ICP–MS	Xu et al. (2015)
Zhenshan 97 × Milyang 46	RILs	243	256	SSR	13.6−44.4	5	4.4–11.7	MR	ICP−OES	Yu et al. (2015)
Xieqingzao × DWR	BILs	202	149	RFLP, SSR	15.08–45.08	6	5.3–11.8	BR/MR	ICP−OES	Hu et al. (2016)
Swarna × Moroberekan	RILs	60	50	SSR	15.82–31.18	3	4–15	BR	AAS	Indurkar et al. (2016)
Nipponbare × *Oryza meridionalis* W1627	BILs	151	164	SSR	–	4	15–21.9	BR	ICP–MS	Ishikawa et al. (2017)
PSBRc82 × Joryeongbyeo	DH	130	6k	SNP	11.28–26.35	7	7.5–16.1	BR/MR	XRF/ICP–MS	Swamy et al. (2018a)
PSBRc82 × IR69428	DH	97	6k	SNP	9.95–23.43	2	20.3–22.8	BR/MR	XRF/ICP–MS	
Swarna × *Oryza nivara* (IRGC81832)	BILs	227	100	SSR	7.1–64.7	7	3–13	BR	AAS	Swamy et al. (2018b)
Swarna × *O. nivara* (IRGC81848)	BILs	245	75	SSR	10.0–25.6	5	10–23	BR	AAS	
MAGIC Plus population	MAGIC	144	14k	SNP	8.1–32	7	9.2–13.7	MR	XRF	Descalsota et al. (2018)
RP‐Bio226 × Sampada	BILs	111	108	SSR	10.6–23.1	3	2.9–34.2	BR	XRF	Dixit et al. (2019)
Goami2 × Hwanseonchal	DH	110	77	SSR	15.30–38.90	14	12.6–46.8	BR	ICPS	Jeong et al. (2020)
Germplasm	Panel	192	31k	SNP	8.2–46.2	17	2.3–47.6	BR/MR	ED‐XRF	Bollinedi et al. (2020)
Germplasm	Panel	102	100	SSR	7.43–27.97	13	3–12	MR	ED‐XRF	Pradhan et al. (2020)
Germplasm	Panel	113	45	SSR	7.3–34.1	2	4.54–5.89	MR	ICP–OES	Islam et al. (2020)
Landraces	Panel	188	148	SSR	14.7–44.3	2	9–14.8	BR	ED‐XRF	Sahu et al. (2020)
IR05F102 × IR69428	DH	148	4.6k	SNP	7.8–26.9	6	8.2–16.2	MR	XRF	IRRI_unpublished
IR95040 × Kaliboro (IRGC77201‐1)	RILs	178	2k	SNP	6.6–37.6	6	6.5–10.6	BR	XRF	IRRI_unpublished
IR95097 × Kaliboro (IRGC 77201‐1)	RILs	195	2.2k	SNP	8.4–37.2	7	5.1–18.3	BR	XRF	
IR14M140 × Jamir (IRGC117765)	RILs	184	7k	SNP	7.31–34.42	5	4–27	MR	XRF	IRRI_unpublished
IR14M141 × Jamir	RILs	297	7k	SNP	9.85–45.91	12	2.3–11.4	MR	XRF	
IR14M125 × Jamir	RILs	200	7k	SNP	11.91–42.31	4	4.5–24.3	MR	XRF	
IR95040 × Jamir	RILs	299	7k	SNP	7.21–43.38	10	1.2–27	MR	XRF	
IR94044 × Jamir	RILs	200	7k	SNP	8.82–37.51	5	3.9–27	MR	XRF	
IR95097 × Jamir	RILs	297	7k	SNP	8.95–41.45	4	2.8–11.8	MR	XRF	
IR14M141 × Kaliboro (IRGC 77201‐1)	RILs	360	7k	SNP	4.75–38.8	8	7.9–21.49	MR	XRF	IRRI_unpublished
IR14M110 × Kaliboro	RILs	120	7k	SNP	9.15–31.8	2	6.9–7.4	MR	XRF	
IR95044:8‐B‐22‐19‐GBS × Kaliboro	RILs	205	7k	SNP	7.05–34.9	3	11.9–20.6	MR	XRF	
IR14M125 × Kaliboro	RILs	210	7k	SNP	5.4–46.3	2	11.5–13	MR	XRF	
MAGIC indica	MAGIC	325	24k	SNP	9.5–22	2	08–09	BR	XRF	IRRI_unpublished

^a^ Type of population: DH, doubled haploid; RILs, recombinant inbred lines; BILs, backcross inbred lines; MAGIC, multiparent advanced generation intercross.

^b^ Population size.

^c^ Number of markers.

^d^ Type of markers: RFLP, restriction fragment length polymorphism; SSRs, simple sequence repeats; SNP, single‐nucleotide polymorphism.

^e^ Range of phenotypic variance of the QTLs.

^f^ Type of rice: BR, brown rice; MR, milled rice.

^g^ Method of assessment of grain Zn: ICP–OES, inductively coupled plasma optical emission spectrometry; AAS, atomic absorption spectrometry; ICP–MS, inductively coupled plasma mass spectrometry; EDXRF, energy‐dispersive X‐ray fluorescence; ICAP, inductively coupled argon plasma spectrometer; ICPS, inductively coupled plasma analyzer; XRF, X‐ray fluorescence.

### 2.2 Consensus map construction and QTL projection

We constructed a consensus map using 2180 SSRs, 1K‐RiCA SNPs, and 7K Infinium SNPs (Arbelaez et al., 2019; IRGSP, 2005; Morales et al., 2020). The missing flanking markers information of the QTLs was retrieved from Gramene and added to the consensus map using their physical positions as a reference (http://www.gramene.org). All the markers used in this study were arranged on their respective chromosomes based on their physical positions. Then the physical positions were converted to cM using the conversion factor 1 cM = 250 kb for building the consensus map (Raghavan et al., 2017). A separate map file was prepared for each chromosome. The QTL and map input files were used for the analysis. The QTL file was projected onto the consensus map to consolidate all the QTL information.

### 2.3 Meta‐QTL analysis

MQTL analysis was performed on the QTL cluster using BioMercator v4.2 (Arcade et al., 2004). We followed the Veyrieras approach for chromosomes with >10 QTLs, and the Goffinet and Garber method for chromosomes with ≤10 QTLs (Veyrieras et al., 2007). The Akaike information criterion (AIC) was used to select the best model for defining the number of MQTLs. Model with the lowest AIC value is regarded as the best model because it corresponds to the least amount of information lost (Akaike, 1978; Sosnowski et al., 2012). The average *R* ^2^ of the initial QTLs was used to calculate the *R* ^2^ of the MQTLs.

### 2.4 Identification of CGs underlying MQTL regions

All the MQTLs were subjected to CG analysis. The physical positions of the markers that flank each MQTL were used to extract the underlying genes within the MQTLs. The batch retrieval option was used to extract all the genes within each MQTL region from the Rice Annotation Project Database (RAP‐DB) (Sakai et al., 2013). Thereafter, genes were filtered based on gene ontology (GO) term or description related to Zn ion binding/transport, Fe ion binding/transport, heme binding, cation ion binding/transport, and metal ion binding, transport, and homeostasis. GenFam was used to determine the enriched gene families among the filtered genes using the options of the Fisher exact test for statistical enrichment, and the Benjamini–Hochberg method to control false discovery rate (Bedre & Madnani, 2019). The higher the –log10(*p*‐value) value, the greater the confidence in the enrichment of the gene family. Finally, 20 CGs involved in Zn or Fe homeostasis were selected for the in silico expression, co‐expression, and haplotype analysis.

### 2.5 Spatiotemporal and co‐expression analyses

RiceXPro v3.0 (https://ricexpro.dna.affrc.go.jp/) was used for the spatiotemporal expression analysis of the selected CGs using the RXP_0001 dataset (Sato et al., 2011). Then the Rice Functionally Related gene Expression Network Database (RiceFREND v2.0) (https://ricefrend.dna.affrc.go.jp/) was used for the construction of co‐expressed gene network for each gene using single guide search (Sato et al., 2013). For the co‐expression analysis, the mutual rank (MR) was set to 10 and the hierarchy to 1. A lower MR value represents stronger co‐expression.

### 2.6 Haplotype analysis of selected CGs across the 3K‐RGP

The selected CGs were subjected to haplotype analysis using the inbuilt tool of the SNP seek database (Mansueto et al., 2017). The “3kfiltered” SNP set representing all the 12 subpopulations was utilized for the analysis (3K‐RGP). Only nonsynonymous or functional SNPs were considered for the analysis. Heterozygotes were considered missing, and then, genotypes with more than 20% missing SNPs were removed before carrying out the analysis. Flapjack 1.22.04.21 was used to visualize the haplotypes of selected genes (Milne et al., 2010).

### 2.7 Identification of superior haplotypes

We used previously generated Zn phenotypic data of *aus* panel at IRRI. These trials were conducted using an alpha lattice design with two replications in 2014WS (Wet Season) and three replications each in 2015WS and 2015DS (Dry Season), with a varying number of blocks in each season. The grain Zn content was determined in polished rice using X‐ray fluorescence (XRF)‐Bruker S2 RANGER. The best linear unbiased estimates (BLUEs) of Zn data for each line were estimated using PBTools v1.4. A total of 134 of these accessions were found to be common between the seasons and were part of the 3K‐RGP. The BLUEs of grain Zn content were used for the phenotypic validation of haplotypes to identify the superior haplotypes (Table S1). The haplotypes found in at least two lines were considered for the analysis. Further, the relationship between Zn content and haplotypes was investigated to identify superior haplotypes. There were lines with more than one superior haplotypes, so haplotype combination effects on grain Zn content were also estimated. The *F*‐statistic and Tukey honestly signficant difference (HSD) tests were used to investigate significant differences among haplotypes and haplotype combinations at *p* < 0.05 (Tukey, 1949).

## 3 RESULTS

### 3.1 Literature search for grain Zn QTLs

**FIGURE 1:**
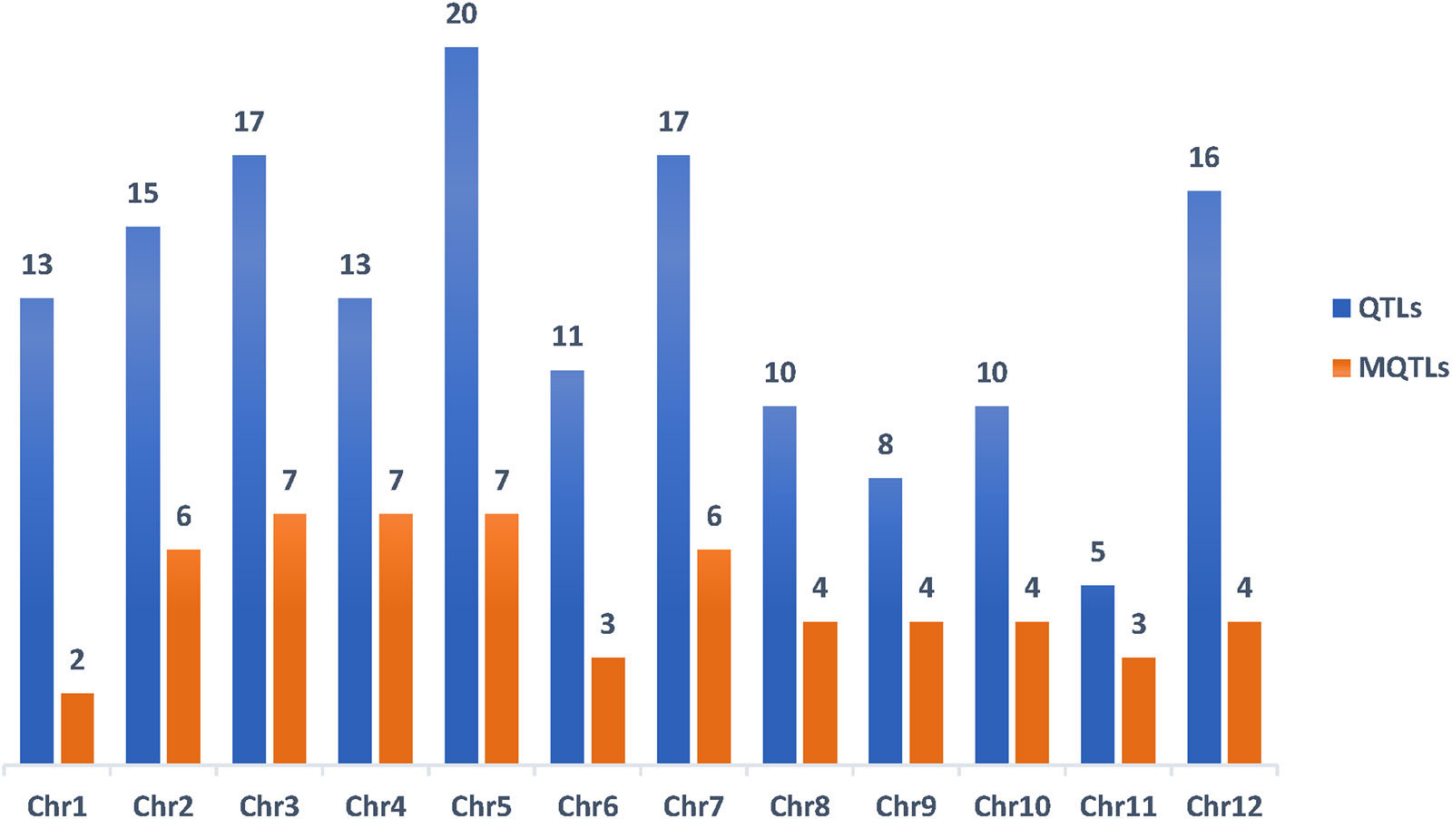
Number of Zn quantitative trait loci (QTLs) projected and meta‐quantitative trait loci (MQTLs) identified on each rice chromosome.

Bibliographic search for the published papers and thesis on Zn QTL mapping identified 26 studies. These included both association mapping and bi‐parental QTL mapping, but majority of the studies were carried out on bi‐parental populations, namely, RILs, BILs DH, and F_2S_, which were developed using elite rice varieties/breeding lines crossed with high Zn donor lines, such as landraces, wild rice, *aus* accessions, and flood‐tolerant rice varieties. The association mapping studies were conducted on multiparent advanced generation intercross (MAGIC) populations and germplasm panels. The Zn content was measured either in brown rice or milled rice or both by using advanced instruments, such as XRF, ICP, and atomic absorption. Predominantly SSRs and SNPs were used for mapping, the number of markers varied from 45 to 31k. Furthermore, the population size ranged from 50 to 378 lines or accessions. The Zn content among the populations ranged from 0.4 to 157.4 ppm. In all 220 Zn QTLs reported, the number of QTLs per study varied from 2 to 17, with 5 or more QTLs identified in 21 studies, and their *R* ^2^ values varied from 0.01% to 47.6% (Table 1). The QTLs lacking physical position information, very low *R* ^2^ values (<1%), and those without overlapping CIs with other QTLs at the same chromosomal region were excluded from the analyses. After careful assessment, a total of 155 Zn QTLs were finalized for further analyses, which were distributed throughout the genome (Figure 1).

### 3.2 Consensus map and MQTL analysis

We constructed a high‐density consensus map using 10,211 markers (2230 SSRs and 7981 SNPs) that included all the flanking makers of 155 Zn QTLs. The consensus map had a length of 372.24 Mb or 1488.9 cM with a marker density of one marker per every 36 kb. As expected, chromosome 1 with 1294 markers had the highest length of 172.9 cM, whereas chromosome 9 with 625 markers had the shortest length of 90.72 cM. On other chromosomes, the number of markers varied from 633 to 1027. Similarly, the map length on other chromosomes also varied from 92.3 to 145.4 cM. Because we used the physical position of the markers, their grouping and order on the chromosomes was accurate, which helped us to precisely project 155 Zn QTLs from different studies on to the consensus map. The number of projected QTLs varied from 5 on chromosome 11 to 20 QTLs on chromosome 5, and in 10 of the 12 chromosomes ≥10 QTLs were distributed (Figure 1).

**FIGURE 2:**
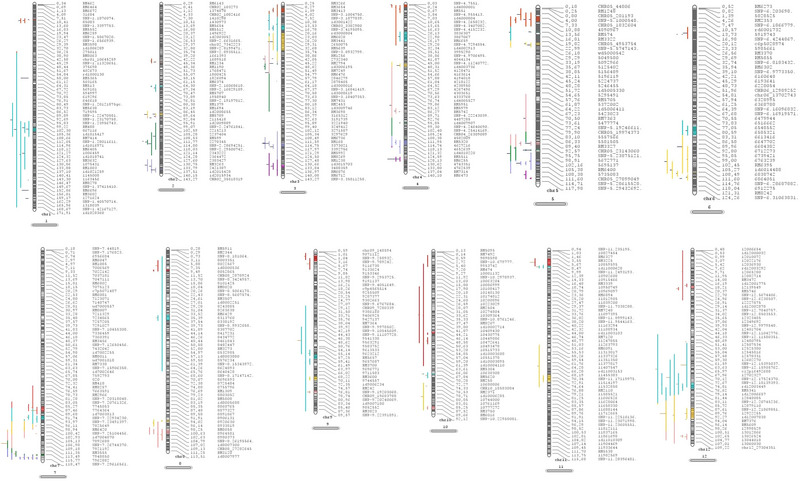
Meta‐quantitative trait loci (MQTLs) identified on various chromosomes of rice for grain Zn. Vertical lines on the left side of each chromosome represents confidence interval (CI) of various quantitative trait loci with colors showing their contribution toward different MQTLs. The filled colored region in chromosome represents the identified MQTLs.

We performed meta‐analyses on QTL clusters chromosome‐wise and used least AIC to declare the number of MQTLs at each of the QTL clusters. A total of 57 MQTLs were identified across all the 12 chromosomes. The MQTLs identified on different chromosomes ranged from 2 to 7. The highest number of seven MQTLs was detected on chromosomes 3–5, whereas lowest two MQTLs were detected on chromosome 1. MQTL_11.1_ and MQTL_11.2_ were found to be overlapping. The number of initial QTLs involved in MQTLs ranged from 2 to 8, but in majority of the MQTLs (49), the initial QTLs involved were 2–4. Four MQTLs (MQTL_1.2_, MQTL_5.4_, MQTL_6.3_, and MQTL_7.2_) were detected from a cluster of five QTLs each on chromosomes 1, 5, 6, and 7, whereas three MQTLs (MQTL_5.1_, MQTL_7.5_, and MQTL_7.6_) involved six initial QTLs on chromosomes 5 and 7, and highest eight QTLs cluster resulted in only one MQTL (MQTL_12.2_) on chromosome 12. The CIs ranged from 0.05 Mb (MQTL_7.4_ and MQTL_10.3_) to 3.7 Mb (MQTL_3.3_). In 26 MQTLs (46%), the CI was ≤300 kb, whereas in 8 MQTLs, it was 300–500 kb, and in the rest of the MQTLs, CI was >500 kb. There was an average 80% reduction in the CI of MQTLs compared to their mean CI of initial QTLs. The *R* ^2^ of MQTLs varied from 2.5% to 45.5%, and 30 (52.6%) MQTLs had *R* ^2^ > 10%. The identified MQTLs are shown in Figure 2, and details of each MQTL are presented in Table 2.

**TABLE 2:** Details of identified meta‐quantitative trait loci (MQTLs) for grain Zn.

MQTL	Chr	AIC	Model	MI^a^	QTLs^b^	PI (bp)^c^	PD (Mb)^d^	CI (cM)^e^	Mean *R* ^2^ (%)^f^
MQTL1.1	1	75.05	2	RM1177‐id1002832	3	3267815–3447298	0.18	0.72–4	8.67
MQTL1.2				RM7202‐905124	5	26197344–26968957	0.77	9.04–132.07	12
MQTL2.1	2	105.71	6	id2005552‐SNP‐2.12329572.	2	12198383–12329577	0.13	0.53–4	8
MQTL2.2				1917475–1927099	2	17283660–17578247	0.29	1.18–6.35	6
MQTL2.3				id2009229‐id2009463	2	23115199–23538743	0.42	1.78–6.19	17.5
MQTL2.4				id2009889‐SNP‐2.24091578.	3	23977679–24097448	0.12	0.59–6.19	12.3
MQTL2.5				RM3212‐2309388	4	28885730–29111475	0.22	0.37–23.4	12.25
MQTL2.6				SNP‐2.34779050.‐SNP‐2.34994149.	3	34784920–35000019	0.22	4–12.01	18
MQTL3.1	3	140.09	7	SNP‐3.876244‐2519506	3	877247–1763294	0.89	3.64–50.97	4.67
MQTL3.2				RM2790‐RM5393	3	4505458–5507754	1.00	4.17–50.97	5.67
MQTL3.3				RM546‐2713838	3	6131773–9795722	3.66	14.78–67.17	19.3
MQTL3.4				2759587‐RM7425	4	11519535–11588760	0.07	0.09–67.17	11.25
MQTL3.5				CHR03_12564300‐2785595	4	12564300–12630582	0.07	0.26–67.17	13.5
MQTL3.6				3392556‐id3014217	3	29473751–30121038	0.65	4.0–65.0	17.5
MQTL3.7				3504185–3517392	3	34072577–34568654	0.50	5.94–65.0	10
MQTL4.1	4	109.04	7	SNP‐4.7551‐3597102	3	8552–603494	0.59	2.64–34.28	4
MQTL4.2				3870434–3959841	2	5802530–7915413	2.11	4.22–34.28	5
MQTL4.3				chr04_12594753‐4146180	2	12594753–13257606	0.66	2.84–72.60	15
MQTL4.4				4475987‐RM6130	3	22737609–22809680	0.07	0.57–72.60	8.67
MQTL4.5				id4008361‐4538469	2	25043240–25214126	0.17	0.69–72.60	5
MQTL4.6				SNP‐4.28661405.‐SNP‐4.29624726.	3	28846553–29809856	0.96	4–72.6	7
MQTL4.7				4745765‐SNP‐4.32845829.	2	32752956–33030943	0.28	4	45.5
MQTL5.1	5	150	7	RM6110‐4882140	5	1493169–2378143	0.88	4–21.40	9.2
MQTL5.2				4928297–4931423	2	4124180–4230799	0.11	0.42–21.40	9
MQTL5.3				4994496–5011602	3	5979409–6374926	0.40	1.72–21.40	9.3
MQTL5.4				RM5311‐5636462	5	22828546–23387450	0.56	3.06–33.08	12.8
MQTL5.5				CHR05_24090514‐5661708	4	24090514–24298176	0.21	0.83–26.48	14.75
MQTL5.6				5713179–5723563	4	26215206–26657752	0.44	2.41–26.48	11.25
MQTL5.7				5780280‐id5014589	2	28831954–29061672	0.23	1.24–5.73	6.5
MQTL6.1	6	72.95	3	RM3408‐5951985	3	4608700–4680281	0.07	0.35–8.11	8.67
MQTL6.2				6585321–6647702	2	20146906–21645145	1.50	6.34–36.26	16.5
MQTL6.3				id6016103‐CHR06_28107682	5	28005249–28107682	0.10	4–36.26	11
MQTL7.1	7	102.14	6	SNP‐7.22188645.‐SNP‐7.22877281.	4	22189639–22878275	0.69	4–32.44	11.5
MQTL7.2				SNP7‐.23491397.‐7824699	5	23492391–23992987	0.50	2.54–32.44	5.4
MQTL7.3				id7004596‐7852517	4	24845026–24939532	0.09	0.19–32.44	5
MQTL7.4				id7004940‐7883795	4	25981825–26031443	0.05	0.26–32.44	12
MQTL7.5				RM1362‐7966459	6	28871504–29079097	0.21	1.0–32.44	12.8
MQTL7.6				SNP‐7.29287104.‐SNP‐7.29385457.	6	29288097–29386450	0.10	1.32–32.44	7.8
MQTL8.1	8	55.98	Model 4	rd8002298‐RM5068	3	2917283–3151554	0.23	0.96–64.62	6.67
MQTL8.2				8331918–8370848	2	9289473–10073191	0.78	3.26–64.62	6.5
MQTL8.3				8935096–8940497	3	24089332–24304409	0.22	0.84–20.64	4.67
MQTL8.4				CHR08_25467581‐8989835	4	25467581–26020138	0.55	2.94–20.64	3.75
MQTL9.1	9	66.25	Model 4	9093193–9125058	2	780446–1635236	0.85	4–6.36	12
MQTL9.2				9649581–9670305	4	14613428–15236075	0.62	1.45–64.60	9.25
MQTL9.3				9753048–9766886	2	17767194–18307070	0.54	2.16–64.60	2.5
MQTL9.4				9809545–9819278	2	19798292–20138665	0.34	1.36–64.60	8
MQTL10.1	10	611.07	Model 4	SNP‐10.11301355.‐SNP‐10.11587437.	3	11372537–11658623	0.29	1.1–57.96	3.3
MQTL10.2				10657139‐RM6704	2	17321074–17490603	0.17	0.65–3.47	10.5
MQTL10.3				SNP‐10.19042282.‐RM7020	2	19113726–19159027	0.05	0.02–16.73	13
MQTL10.4				10792894‐RM591	2	21980330–22451229	0.47	1.87–16.73	10
MQTL11.1	11	22.65	Model 3	10858811‐id11000778	3	1378959–2514115	1.14	1.74–4.54	16.3
MQTL11.2				chr11_2393690‐SNP‐11.2754574.	3	2393690–2758671	0.36	1.74–4.54	16.3
MQTL11.3				11499828‐RM457	2	17908099–18864049	0.96	4.0–24.0	7
MQTL12.1	12	117.29	4	id12002362‐12179231	3	5170392–5566618	0.40	1.71–28.46	10
MQTL12.2				SNP‐12.18906426.‐12827606	8	18934992–19829588	0.89	4.0–50.61	16.62
MQTL12.3				12926167–12981598	3	22544640–24043382	1.50	6.11–50.61	23.3
MQTL12.4				13052337‐chr12_26665728	4	26471755–26665728	0.19	1.53–30.6	20

Abbreviations: AIC, Akaike information criterion; CI, confidence interval.

^a^ Flanking markers of the MQTL.

^b^ Number of corresponding initial QTLs of the MQTLs.

^c^ Physical positions of the flanking markers of the MQTLs in basepair (bp).

^d^ Physical interval of the MQTLs in megabasepair (Mb).

^e^ Range of CI of corresponding initial QTLs of the MQTLs in centimorgans (cM).

^f^ Mean phenotypic variance of the corresponding initial QTLs.

### 3.3 Mining of CGs underlying MQTL regions

A total of 4196 genes within MQTL regions were obtained from RAP‐DB. Following filtering based on the functional role and GOs, 318 genes were selected within 51 MQTLs (Table S2). Based on GO, 35.8% of genes were associated with Zn ion binding and Zn ion transport, 14.2% with Fe ion binding and transport, 9.7% with cation binding and transport, 9.7% with metal ion binding and transport, 6.3% with heme binding, and 18.2% genes were enriched with oxidation–reduction process, cell redox homeostasis, electron carrier activity, and transmembrane transport. A new computational approach, GenFam allows annotation, classification, and enrichment of genes based on their gene family. Among 318 genes identified, 190 genes were annotated and classified into gene families by GenFam, resulting in 60% interaction/coverage with the GenFam database. A total of 49 gene families were identified with 23 of them being overrepresented (*p*‐value <0.05). The most enriched gene family was the RING finger domain gene family followed by Cytochrome P450. The other enriched families include the Zn finger gene family, copper chaperone gene family, glycoside hydrolase gene family, and 2OG‐Fe(II) oxygenase gene family (Table S3 and Figure S1).

Among the identified CGs, 20 genes involved in Zn and Fe homeostases were chosen for expression, co‐expression, and haplotype analysis. These genes were found on 11 different MQTLs distributed on chromosomes 3–7, 11, and 12. The genes are from the ZIP family (*OsZIP5*, *OsZIP7*, and *OsZIP9*), the mugineic acid family phytosiderophores (*OsSAMS1*, *OsDMAS1*, *OsNAS3*, *OsTOM1*, *OsTOM2*, and *OsTOM3*), metal transporters (*OsFRO1*, *OsMTP1*, *OsMTP7*, and *OsFRDL1*), metal‐related genes (*OsNRAMP7*), transcription factor for Fe homeostasis (*OsbHLH133*), small GTPase for Fe homeostasis (*OsRab6B2*), and metallothioneins (*OsMT1f*, *OsMT1a*, *OsMT1g*, and *OsMTId*). The details of selected genes are presented in Table 3.

**TABLE 3:** List of selected candidate genes and their chromosomal distribution.

MSU ID	RAP ID	Gene symbol	Chr	Position (kb)	MQTL	Description/Function	References
*LOC_Os03g09140*	*Os03g0191400*	*OsRab6B2*	Chr3	4763.79–4766.34	MQTL3.2	Small GTPase, regulation of iron homeostasis	Yang and Zhang (2016)
*LOC_Os03g11734*	*Os03g0216700*	*OsFRDL1*	Chr3	6131.85–6142.79	MQTL3.3	Citrate transporter, efficient translocation of Fe	Yokosho et al. (2009)
*LOC_Os03g13390*	*Os03g0237100*	*OsDMAS1*	Chr3	7228.96–7231.75	MQTL3.3	Similar to NADPH‐dependent codeinone reductase, synthesis of mugineic acid	Bashir et al. (2006) and Aung et al. (2019)
*LOC_Os04g23180*	*Os04g0298200*	*OsMTP7*	Chr4	13176.75–13183.06	MQTL4.3	Similar to metal tolerance protein	Chen et al. (2013) and Ram et al. (2019)
*LOC_Os04g48930*	*Os04g0578600*	*OsFRO1*	Chr4	29178.86–29181.67	MQTL4.6	Ferric reductase oxidase, iron homeostasis, “drought, heat, salinity stress response”	Banerjee and Chandel (2011), Gross et al. (2003); Muhammad et al. (2018)
*LOC_Os05g03780*	*Os05g0128400*	*OsMTP1*	Chr5	1675.5–1679.05	MQTL5.1	Cation diffusion facilitator (CDF) family protein, translocation of Zn, Cd, and other heavy metals	Menguer et al. (2013)
*LOC_Os05g04510*	*Os05g0135700*	*OsSAMS1*	Chr5	2089.03–2092.34	MQTL5.1	*S*‐Adenosyl‐l‐methionine synthetase, mugineic acid biosynthesis	Widodo et al. (2010)
*LOC_Os05g10940*	*Os05g0198400*	*OsZIP7*	Chr5	6090.8–6094.14	MQTL5.3	Plasma membrane Zn transporter of the Zn regulated; iron‐regulated transporter‐like protein (ZIP) family	Yang et al. (2009a) and Yang et al. (2020)
*LOC_Os05g39540*	*Os05g0472400*	*OsZIP9*	Chr5	23204.53–23213.28	MQTL5.4	Influx transporter for Zn and Cd, zinc/cadmium uptake	
*LOC_Os05g39560*	*Os05g0472700*	*OsZIP5*	Chr5	23216.41–23219.28	MQTL5.4	Influx transporter for Zn and Cd, zinc/cadmium uptake	
*LOC_Os07g48980*	*Os07g0689600*	*OsNAS3*	Chr7	29323.09–29324.72	MQTL7.6	Nicotianamine synthase 3, mitigation of excess Fe stress, Fe detoxification, and transport of iron	Inoue et al. (2003) and Aung et al. (2019)
*LOC_Os11g04020*	*Os11g0134900*	*OsTOM1*	Chr11	1618.49–1623.98	MQTL11.1	Efflux transporter of deoxymugineic acid, phytosiderophore efflux transporter, iron acquisition	Nozoye et al. (2015, 2011) and Ricachenevsky et al. (2011)
*LOC_Os11g04030*	*Os11g0135000*	*OsTOM2*	Chr11	1626.49–1631.41	MQTL11.1	Efflux transporter of phytosiderophores, metal transport, similar to major facilitator superfamily antiporter	
*LOC_Os11g04104*	*Os11g0135900*	*OsTOM3*	Chr11	1657.87–1665.11	MQTL11.1	Homolog of transporter of mugineic acid family phytosiderophores 1 (TOM1) similar to carbohydrate transporter/sugar porter/transporter	
*LOC_Os12g32400*	*Os12g0508500*	*OsbHLH133*	Chr12	19546.56–19547.56	MQTL12.2	bHLH transcription factor, regulation of iron distribution	Wang et al., (2013), Bashir et al. (2014)
*LOC_Os12g38010*	*Os12g0567800*	*OsMT1f*	Chr12	23352.19–23353.19	MQTL12.3	Metallothionein, Zn/Fe homeostasis	Aung et al. (2018), Yang et al. (2009b), and Kim and Kang (2018)
*LOC_Os12g38270*	*Os12g0570700*	*OsMT1a*	Chr12	23501.36–23502.39	MQTL12.3		
*LOC_Os12g38290*	*Os12g0571000*	*OsMT1g*	Chr12	23512.5–23513.48	MQTL12.3		
*LOC_Os12g38300*	*Os12g0571100*	*OsMT1d*	Chr12	23515.15–23515.8	MQTL12.3		
*LOC_Os12g39180*	*Os12g0581600*	*OsNRAMP7*	Chr12	24119.87–24123.72	MQTL12.3	Similar to integral membrane protein, transmembrane transport of Zn	Sperotto et al. (2010)

### 3.4 Expression profile and co‐expression network of selected CGs

For all the genes except *OsMTP7* and *OsbHLH133*, expression information was available in the expression database (RXP_0001). The heat map shows the time of sampling, the amount of tissue sampled for expression analysis, and the level of expression of different genes in 12 different vegetative and reproductive tissues at different stages of their development (Figure S2). Most of the genes except *OsMTP1* and *OsNRAMP7* were differentially expressed with moderate‐to‐high level of transcripts in both vegetative and reproductive tissues. However, they were predominantly expressed in leaf blades, leaf sheaths, and roots compared to reproductive tissues. Some of the genes were highly expressed in stem (*OsFRDL1*, *OsZIP5*, and *OsMT1a*), ovary (*OsFRDL1*, *OsDMAS1*, *OsNAS3*, and *OsTOM2*), inflorescence (*OsFRO1*), anther (*OsRab6B2*, *OsDMAS1*, *OsFRO1*, *OsMT1a*, and *OsMT1g*), and pistil (*OsZIP5*). Similarly, *OsZIP5* is expressed in roots, leaf blade, leaf sheath, stem, inflorescence, pistil, lemma, palea, and ovary. However, *OsTOM1* was expressed in roots and leaf sheath, *OsTOM2* expressed in ovary, and in almost all the stages of embryo and endosperm development, including roots and leaf sheath. High expression was also observed in endosperm for *OsRab6B2*, *OsDMAS1*, *OsMT1f*, and *OsMT1g*.

Results of the co‐expression network analyses are presented in Figure S3. *OsNAS3* was found to co‐express with *OsNAAT2* and other genes involved in oxidoreductase activity and abiotic and biotic stress tolerances. *OsDMAS1* co‐expressed with *OsMTK2*, a gene involved in methionine biosynthesis. *OsSAMS1* co‐expressed with *LOC_Os12g42876*, a gene having Zn binding function, as well as other genes that control shoot branching, nitrogen use efficiency, and the synthesis of secondary metabolites. The *OsNRAMP1*, *OsIRO2*, *OsIMA1*, and *OsIMA2* involved in Fe homeostasis were co‐expressed with *OsFRO1*. *OsZIP5* displayed co‐expression with trehalose biosynthetic gene. The genes associated with signal transduction and gluconeogenesis co‐expressed with *OsZIP7*. Zn transporters *OsZIFL9* and *OsTOM1* co‐expressed. Similarly, *OsTOM2* and *OsTOM3* co‐expressed with Zn transporters *OsZIFL10* and *OsZIFL12*, respectively, as well as with other genes involved in cellular metabolic processes and oxidoreductase activity. The co‐expressed genes of *OsMT1f* were associated with copper stress, salt stress, and pathogen infection. In the case of *OsMT1d*, the co‐expressed genes like *OsSa1T*, *OsHAC4*, *OsEXPB2*, and *OsMT1Ld* were related to salt tolerance, arsenic tolerance, root system architecture, and metallothionein, respectively, whereas *OsMT1a* also showed co‐expression with *OsSa1T*. Regarding the co‐expression network of *OsRab6B2*, genes regulating biotic stress tolerance, carbohydrate metabolism, and transmembrane transport were present.

### 3.5 Identification of superior haplotype of selected CGs

**FIGURE 3:**
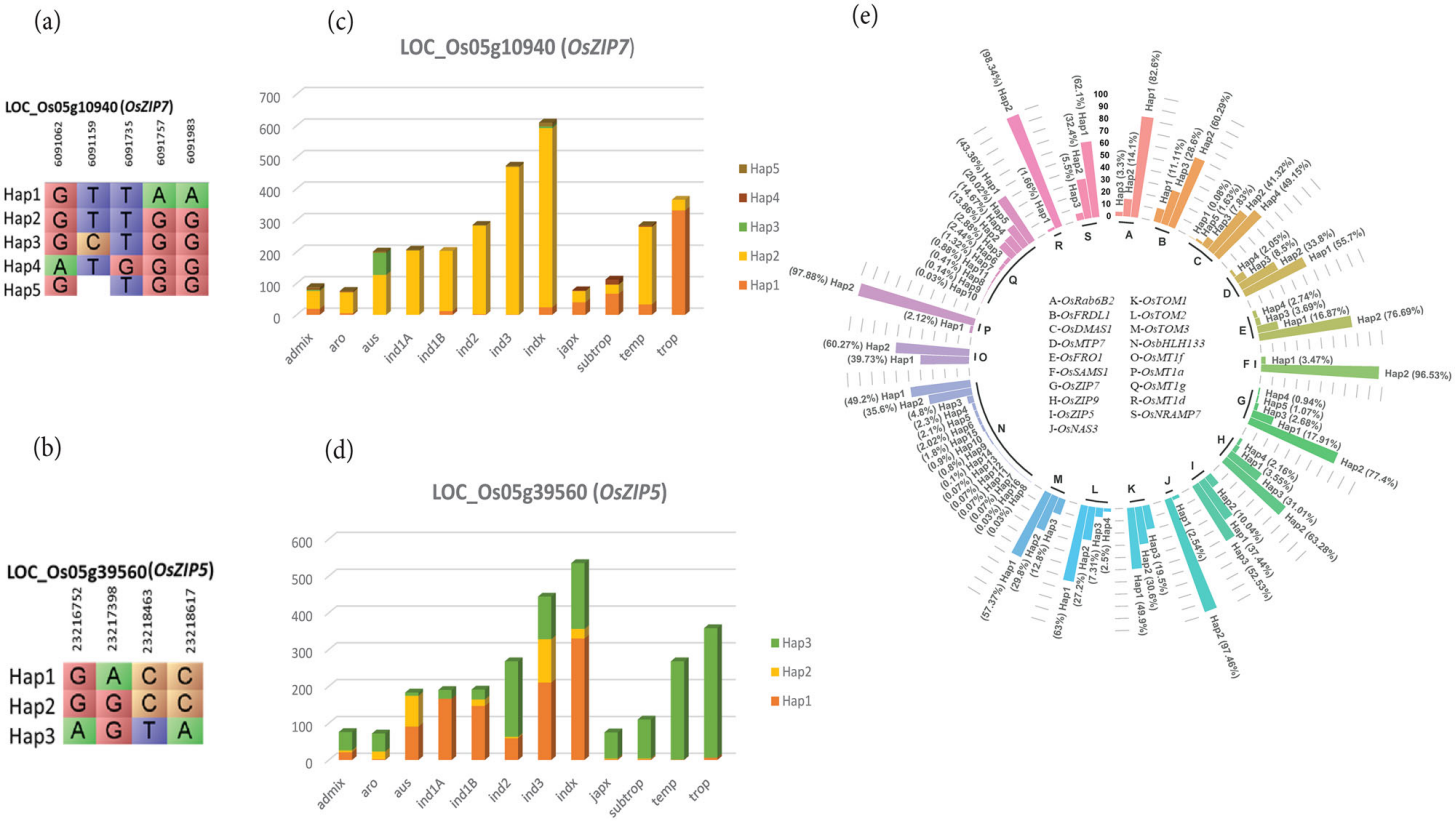
Parts (a) and (b) represent the haplotypes identified across 3K rice genome panel (3K‐RGP) for *OsZIP7* and *OsZIP5*, respectively; parts (c) and (d) show the distribution of haplotypes in different subpopulations within 3K RG panel for the respective genes; and (e) circular bar chart showing frequency of haplotypes within various selected genes.

Haplotypes were identified for the selected 20 CGs using the rice SNP seek database representing 3K‐RGP. The *OsMTP1* did not reveal any haplotype. For the remaining genes, the number of haplotypes varied from 2 to 16 (Figure 3a,b and Figure S4). Two haplotypes for 5 genes, 3 haplotypes for 6 genes, 4 haplotypes for 4 genes, 5 haplotypes for 2 genes, and 11 and 16 haplotypes for 1 gene each were identified from 3K‐RGP. The frequency of haplotypes of the selected genes is presented in Figure 3e.

**FIGURE 4:**
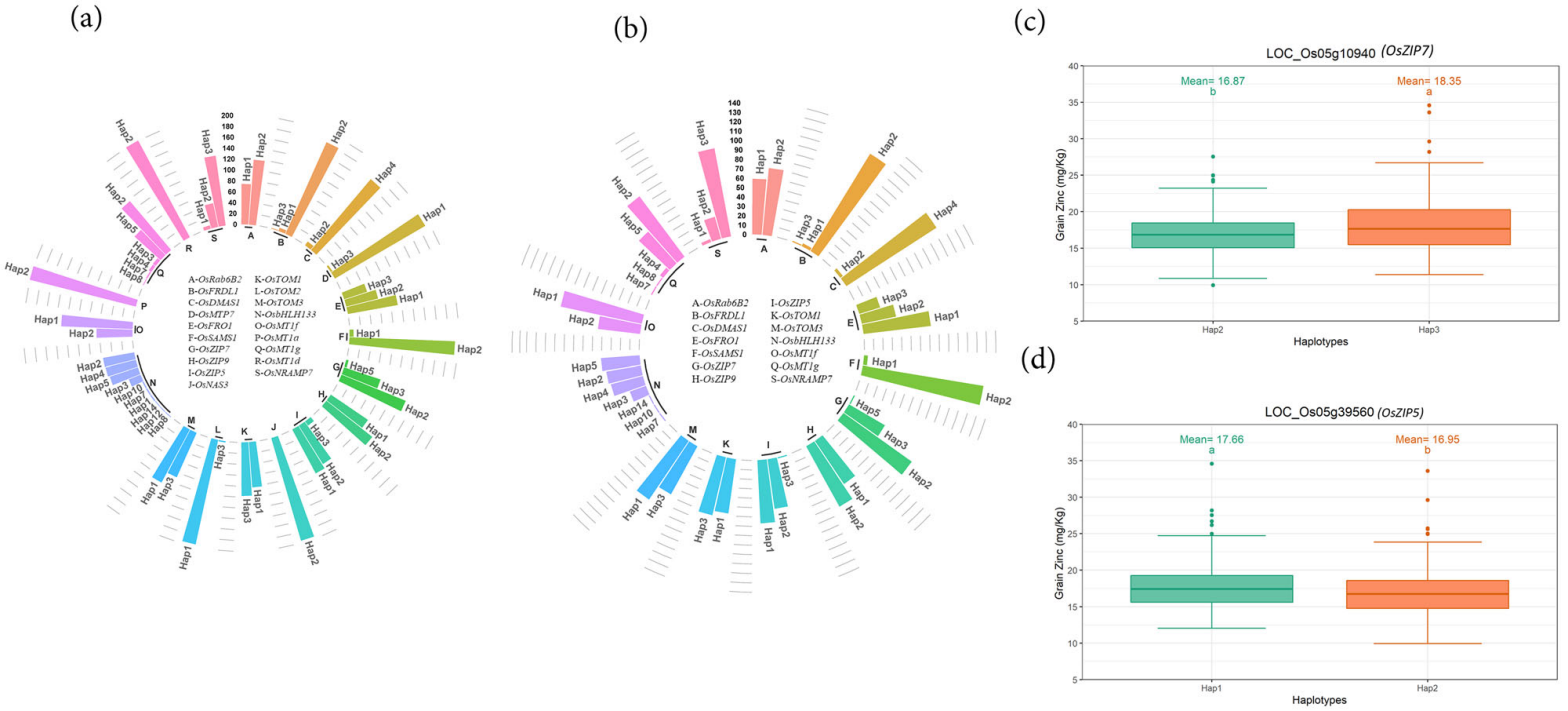
(a) Haplotypes distribution of various genes in *aus* subpopulation of 3K rice genome panel (3K‐RGP); (b) haplotype distribution in 134 lines of *aus* subpopulation for the genes used for phenotypic validation; parts (c) and (d) represent the range of Zn content in 134 lines of *aus* subpopulation across three seasons for haplotypes of *OsZIP7* and *OsZIP5*, respectively. Different alphabets denote statistically significant differences and vice versa from Tukey honestly significant difference test.

We carried out the validation of haplotypes and haplotype combinations using the Zn data of 134 *aus* accessions evaluated for three seasons at IRRI. The range of Zn content of various haplotypes across three seasons is presented in Table S4. However, five genes had only one haplotype and were removed before conducting the analysis (Figure 4a). Finally, 14 genes were selected for the analysis, out of which 5 genes did not show any significant haplotype effects (Figure 4b). The haplotypes of nine genes were further tested by mean comparison using the Tukey HSD post hoc test, and superior haplotypes were identified (Table 4). The superior haplotypes with highest Zn content were Hap1 for *OsZIP5* (17.66 mg/kg), *OsNRAMP7* (21.17 mg/kg), and *OsMT1f* (17.63 mg/kg); Hap2 for *OsRab6B2* (18.13 mg/kg), *OsDMAS1* (19.77 mg/kg), and *OsZIP7* (18.35 mg/kg); Hap3 for *OsZIP9* (17.77 mg/kg) and *OsFRO1* (18.35 mg/kg), and Hap4 for *OsMT1g* (21.92 mg/kg), respectively (Figure 4c,d and Figure S5). The Zn content of the lines increased with an increase in the number of superior haplotypes, among the six superior haplotype combinations for nine genes, lines with five haplotypes (20.6 mg/kg) were significantly different from all the other groups, whereas there was a nonsignificant decrease in the Zn content of lines with six superior haplotypes over lines with five superior haplotypes (Table S5).

**TABLE 4:** Haplotype‐Phenotype relationship for the genes associated with grain Zn.

Gene	Hap1	Hap2	Hap3	Hap4	Hap5	Superior haplotypes
*LOC_Os03g09140 (OsRab6B2)*	16.49 (b)	18.13 (a)	–			Hap2
	*n* = 59	*n* = 71				
*LOC_Os03g13390 (OsDMAS1)*		19.77 (a)		17.3 (b)		Hap2
		*n* = 3		*n* = 114		
*LOC_Os04g48930 (OsFRO1)*	17.41 (ab)	16.69 (b)	18.35 (a)			Hap3
	*n* = 72	*n* = 37	*n* = 23			
*LOC_Os05g10940 (OsZIP7)*		16.87 (b)	18.35 (a)		NV	Hap3
		*n* = 89	*n* = 44			
*LOC_Os05g39540 (OsZIP9)*	16.79 (b)	17.77 (a)				Hap2
	*n* = 55	*n* = 73				
*LOC_Os05g39560 (OsZIP5)*	17.66 (a)	16.95 (b)	NV			Hap1
	*n* = 67	*n* = 53				
*LOC_Os12g38010 (OsMT1f)*	17.63 (a)	16.77 (b)				Hap1
	*n* = 88	*n* = 45				
*LOC_Os12g38290 (OsMT1g)*		17.28 (b)	–	21.92 (a)	17.03 (b)	Hap4
		*n* = 80		*n* = 4	*n* = 45	
*LOC_Os12g39180 (OsNRAMP7)*	21.17 (a)	17.94 (b)	17.21 (b)			Hap1
	*n* = 3	*n* = 24	*n* = 95			

*Note*: *n*: number of accessions; –: absent in 134 selected lines; NV: not validated. Different alphabet denotes statistically significant differences and vice versa.

The distribution of superior haplotypes of the above‐discussed nine genes varied in different subpopulations of 3K‐RGP, and some of them were predominantly present in certain groups (Figure 3c,d and Figure S6). The superior haplotypes for *OsZIP7*, *OsFRO1*, and *OsRab6B2* were found in high frequency in *aus* subgroup, *OsZIP9* and *OsDMAS1* in *indica*, whereas superior haplotypes for *OsZIP5* and *OsMT1f* were found in *aus* and *indica*, whereas for *OsMT1g* in *japonica*, *subtropical*, *temperate*, *tropical*, and *aromatic* subgroups, whereas superior haplotype for *OsNRAMP7* was distributed across the subgroups with low frequency in *aus*.

## 4 DISCUSSION

Biofortification of rice with high grain Zn content is one of the feasible options to address Zn malnutrition in rice‐consuming Asian countries (Bouis & Saltzman, 2017; Kassebaum et al., 2014; Nestel et al., 2006; Wessells et al., 2012). Several conventional bred high Zn rice varieties have been released for commercial cultivation but the efforts are targeted, isolated, and the whole process has been slow (Calayugan et al., 2021; Inabangan‐Asilo et al., 2019). Mainstreaming of grain Zn trait as an important component of all the future rice varieties helps to address Zn malnutrition on a larger scale (Palanog et al., 2019). In our study, we carried out MQTL analyses of Zn QTLs, shortlisted candidate metal homeostasis genes, and identified superior haplotypes to support mainstreaming the Zn biofortification of rice.

It is important to note that >200 QTLs for grain Zn and Fe have been reported in rice (Raza et al., 2019). If this valuable public genomics resource is processed and used in Zn breeding, it has the potential to enhance the efficiency of rice Zn biofortification (Swamy et al., 2016). MQTL analyses is a popular, user‐friendly, and in silico approach for the consolidation of all the QTLs for a trait, and identification of MQTLs with stable expression and narrower CIs enables their use in MAB and GS (Arcade et al., 2004; Goffinet & Gerber, 2000; Gong et al., 2018; Norton et al., 2008; Swamy et al., 2016). As a result, a number of MQTL studies were carried out for various traits in multiple crops reporting significant reduction in redundant QTLs and their CIs (Guo et al., 2018; Islam et al., 2019; Khahani et al., 2020; Soriano et al., 2021; Swamy et al., 2011; Selamat & Nadarajah, 2021; Saini et al., 2022; Sheoran et al., 2022). But it is worth noting that most of these studies used sparse linkage maps with different types of markers, resulting in a lack of common markers among studies and with that of reference map leading to difficulties in QTL consolidation, to accurately locate MQTLs and to precisely narrow down their CIs (Cai et al., 2019; Jin et al., 2015; Raza et al., 2019; Zhang et al., 2017). In two previous MQTL studies on grain Zn content in rice as well sparse linkage maps were used (Jin et al., 2015; Raza et al., 2019). Therefore, the use of high‐density linkage maps is useful in identifying stable MQTLs with much narrower CIs for use in breeding and in prioritizing CGs for functional characterization.

We used genetic studies conducted on grain Zn content in rice utilizing mainly SNPs and SSRs. We also constructed a high‐density consensus map that consisted of 10,211 markers (SNPs and SSRs) with an average marker interval of 0.14 cM. The meta‐analyses of 155 Zn QTLs summarized them to 57 MQTLs with a significant reduction of 63.2% in Zn QTLs, which suggests that most of the previously mapped Zn loci were shared by multiple populations. The physical intervals of 15 and 19 MQTLs identified in the current study matched with the MQTLs reported in the prior 2 studies, respectively, and 5 of these MQTLs were shared by all 3 studies (Jin et al., 2015; Raza et al., 2019) (Table S6). The CIs were also reduced on average by 80%, which is higher than achieved in previous studies, thus improving the mapping resolution of the genomic regions. The CIs of MQTLs ranged from 0.05 to 3.66 Mb, whereas they ranged from 0.07 to 11.64 Mb in previous reports (Jin et al., 2015; Raza et al., 2019). In our study, 46% of the MQTLs had CI < 300 kb, 14% had 300–500 kb, 29% had 500 kb–1 Mb, and 10.5% had >1 Mb. In previous study by Raza et al. (2019), only 8.6% MQTLs had <300 kb, 8.7% with 300–500 kb, 21.7% with 500 kb–1 Mb, and 61% had >1 Mb, whereas all the MQTLs except one locus had CI > 1 Mb in another study (Jin et al., 2015). All the common MQTLs except MQTL3.3 had narrower CIs in our study. The *R* ^2^ of MQTLs varied from 2.5% to 45.5% with 52.6% of the MQTLs having >10% *R* ^2^. Further, five MQTLs (MQTL_1.2_, MQTL_5.4_, MQTL_6.3_, MQTL_7.5_, and MQTL_12.2_) were found to be some of the interesting MQTLs based on the following criteria: average phenotypic variance >10% and involvement of a large number of initial QTLs within the MQTL. These MQTLs have the potential to be the most effective candidate targets for use in marker‐assisted selection (MAS) after being validated in various genetic backgrounds and environments utilizing linked flanking markers. The MQTLs identified in this study provide more closely linked markers for use in GAB to improve grain Zn content in rice.

The results of GO and GenFam analyses of genes co‐located with MQTLs showed that these genomic regions were heavily enriched with metal homeostasis genes (94.1%) having diverse functions. Some of the major gene families are zinc finger, RING Finger, Cytochrome P450, Glycoside hydrolase, and Fe‐oxygenase genes (Table S3). The Zn finger and RING finger motifs are important transcription factors that play role in plant stress regulation (Rhodes & Klug, 1993; Vallee & Falchuk, 1993; Kiełbowicz‐Matuk, 2012). Cytochrome P450 family genes are vital for detoxification, metal homeostasis, and plant growth and development (Aung et al., 2018; Bandyopadhyay et al., 2017). However, Fe oxygenase family genes help Fe metabolism under Fe‐deficient conditions (Nakanishi et al., 2000; Vigani et al., 2013). Similarly, glycoside hydrolase and copper chaperone gene families have also been found to be regulated under Zn, Fe, or heavy metal stress (Quinet et al., 2012; Zeng et al., 2019). High concentration of metal homeostasis genes/gene families, such as *OsFER*, *OsFRDL*, *OsMTP6*, *OsMT2D*, *OsNAS1*, *OsNAS2*, *OsNAS3*, *OsNRAMP7*, *OsVIT1*, *OsYSL*, *OsZIFL*, and *OsZIP*, with Fe and Zn QTLs have been previously reported in MAGIC Plus, Global MAGIC, and doubled haploid populations of rice (Descalsota et al., 2018; Swamy et al., 2018a; Swamy et al., 2018b; Calayugan et al., 2020; Zaw et al., 2019).

However, a deeper insight into the MQTLs revealed that at least 20 major metal homeostasis genes were associated with 11 MQTLs (Table 3). Most of the genes were differentially expressed in both vegetative and reproductive tissues. Overexpression of *OsNAS3* in roots and leaf blades in rice has been previously reported (Inoue et al., 2003). Likewise, the expression of *OsTOM2* in endosperm and *OsTOM1* in roots is comparable to that previously described (Ricachenevsky et al., 2011). The majority of these genes co‐expressed with other metal homeostasis genes, genes involved in growth and development, and biotic and abiotic stress tolerances. *OsNAAT2* was found to be strongly co‐expressed with *OsNAS3* suggesting its role in Fe/Zn uptake. In similar manner, the co‐expressions of *OsFRO1* with *OsNRAMP1*, *OsIRO2*, *OsIMA1*, and *OsIMA2* suggest their coordinated involvement in Fe/Zn homeostasis. *OsMTK2*, a 5‐methylthioribose (MTR) kinase gene, has role in Met (Methionine) cycle (Sauter et al., 2004). According to the study by Aung et al. (2018), *OsDMAS1* and *OsMTK2* were both found to be suppressed in roots under Fe toxicity, and it was also suggested that the Met cycle might play a role in nicotianamine synthesis under excess Fe. Similarly, *OsTOM1* was found to be co‐expressed with *OsZIFL9*, *OsTOM2* with *OsZIFL10*, and *OsTOM3* with *OsZIFL12*, probably due to their high similarity (Ricachenevsky et al., 2011). However, more confirmatory research is needed to validate and comprehend the co‐expression network, which could lead to new insights into plant responses to nutrient deficiency. There have been several earlier studies on physiological, molecular, and transgenic validations of these genes proving their role in the production of root exudates, chelation, and minerals uptake, transport, partitioning, and loading into grains in rice (Table 3). It clearly shows that micronutrient movement from soil to grain is a complex process, which involves a coordinated function of the multiple metal homeostasis genes at different stages of crop growth, especially during reproductive stage to determine the grain mineral content of rice varieties (Sperotto et al., 2014; Swamy et al., 2016; Waters et al., 2012; Xia et al., 2021, 2020).

Identification of haplotypes for different genes and their use in crop improvement through allele mining, pyramiding, or genomic selection is found to be efficient in achieving better genetic gains (Abbai et al., 2019; Addison et al., 2020; Anandan et al., 2022; Bhat et al., 2021; Du et al., 2022). Using genome editing approach, two yield‐related genes *Gn1a* and *DEP1* were altered and genome‐edited lines revealed that one mutant allele of *Gn1a* and three of *DEP1* provides higher yield as compared to other natural high‐yield alleles (Huang et al., 2018). We identified 2–16 haplotypes for 19 metal homeostasis genes in 3K‐RGP, and their distribution varied across the subgroups. We were able to phenotypically validate haplotypes of 14 genes in a subset of *aus* lines and identified superior haplotypes for 9 genes (Figure 4b, Table 4). The frequencies of superior haplotypes varied in different subpopulations but were predominantly present in certain groups (Figure S6). The superior haplotypes for *OsZIP7*, *OsFRO1*, *OsRab6B2*, *OsZIP5*, and *OsMT1f* were in high frequency in *aus* and *indica* subgroups, *OsZIP9* and *OsDMAS1* in *indica*, whereas *OsMT1g* in *japonica* and *aromatic* subgroups. Previous research has also found that haplotype distribution is determined by evolutionary and population genetic factors such as mutation and recombination rates, as well as selection (Magwa et al., 2016; Zaitlen et al., 2005). It provides an idea to the breeders for the selection of best donors for their trait of interest from the gene bank. For example, *aus* accessions were found to be a rich source of micronutrients, and several high Zn donor accessions, such as Kaliboro (IRGC 77201‐1), Jamir (IRGC117765), Lalsaita (IRGC 43915‐1), and UCP122 (IRGC 8794‐1), have been identified and extensively used in the Zn biofortification program at IRRI (Calayugan et al., 2021; Palanog et al., 2019; Swamy et al., 2016).

The superior haplotypes with highest Zn content were Hap1 for *OsZIP5*, *OsNRAMP7*, and *OsMT1f*; Hap2 for *OsRab6B2*, *OsDMAS1*, and *OsZIP7*; Hap3 for *OsZIP9* and *OsFRO1*, and Hap4 for *OsMT1g*, respectively (Figure 4c,d and Figure S5). On overall bases, superior haplotypes of each gene added 2 mg/kg of Zn. Superior haplotypes, Hap‐CA (*qZn_5.1_*) and Hap‐GC (*qZn_12.1_*), enhanced the Zn content in a MAGIC plus population. Similarly, Hap‐AT (*qZn_6.2_*) and Hap‐CAGTTT (*qZn_7.1_*) increased the Zn content in doubled haploid populations of rice (Descalsota et al., 2018; Calayugan et al., 2020). It is worth noting that in this study, the Zn content of the lines increased with an increase in number of superior haplotypes; the group of lines with five superior haplotypes was significantly different from all the other groups and had a Zn increment of 2.5 mg/kg (Table S5). Previous studies showed that the pyramiding of QTL superior alleles improved the seed storability (Wu et al., 2021). In the same way, haplotype analysis of 21 yield‐ and grain‐quality–related genes in rice showed that lines with multiple superior haplotypes performed better (Abbai et al., 2019). Overall, haplotype‐based breeding has a huge potential for developing tailor made rice varieties with improved yield, grain quality, and nutrition. The superior haplotype‐specific markers would be utilized in order to combine them into single background as well as to choose the ideal combination of haplotypes controlling the particular phenotype. However, the robust validation of haplotypes, selection of the right combination of haplotypes, understanding the environmental impact, haplotype and environment interactions, pleiotropic effects, and interactions among haplotypes are prerequisite for the success of a haplotype‐based breeding program (Abbai et al., 2019; Mayer et al., 2020).

## 5 CONCLUSION

MQTL analysis is a popular in silico approach to identify precise and consistent genomic regions for use in breeding and to prioritize CGs for functional validation. In the present study, MQTL analyses of 155 Zn QTLs using a high‐density genetic map identified 57 MQTLs, with a significant reduction in QTLs (63.2%) and their CIs (80%). MQTLs were found to be enriched with metal homeostasis genes/gene families with diverse functions indicating their importance in metal uptake, transport, partitioning, and loading into the grains. In silico differential expression and co‐expression analyses revealed a complex network of genes involved in metal homeostasis. The CG haplotypes analyses using 3K‐RGP showed variations with their predominance in certain subgroups, and superior haplotypes were identified using Zn data of a subset of *aus* lines. On an average superior haplotype contributed 2 mg/kg more Zn and a group of lines with five superior haplotypes performed better over the others. The MQTLs, CGs, and superior haplotypes identified in our study are useful for the efficient Zn biofortification of rice.

## AUTHOR CONTRIBUTIONS

**Gaurav Joshi**: Formal Analysis; Writing‐original draft. **Yan Paing Soe**: Investigation. **Alvin Palanog**: Investigation; Formal Analysis. **Tapas Kumer Hore**: Formal Analysis. **Chau Thanh Nha**: Investigation; Formal Analysis. **Mark Ian Calayugan**: Investigation; Formal Analysis. **Mary Ann Inabangan‐Asilo**: Project administration. **Amery Amparado**: Project administration. **Indra Deo Pandey**: Writing‐review & editing. **Pompe C. Sta Cruz**: Writing‐review & editing. **Jose E. Hernandez**: Writing‐review & editing. **B. P. Mallikarjuna Swamy**: Conceptualization; Methodology; Supervision; Writing‐review & editing.

## CONFLICT OF INTEREST STATEMENT

The authors declare that the research was conducted in the absence of any commercial or financial relationships that could be construed as a potential conflict of interests.

## Supporting information

FIGURE S1 Graphical summary of GenFam enrichment analysis.
FIGURE S2 Spatiotemporal expression analysis of selected genes using RiceXPro.
FIGURE S3 Co‐expression network of the selected genes.
FIGURE S4 Haplotypes identified for various genes using 3K‐RG panel.
FIGURE S5 Boxplots showing range of zinc content among various haplotypes across three seasons.
FIGURE S6 Distribution of haplotypes of various genes across different subpopulations in 3K‐RG panel.

TABLE S1 BLUEs of 134 *aus* lines for phenotypic validation of haplotypes.
TABLE S2 Genes underlying identified MQTLs.
TABLE S3 GenFam enrichment analysis.
TABLE S4 Range of zinc content among various haplotypes across three seasons.
TABLE S5 Mean comparison of Zn in *aus* lines group based on number of superior haplotypes.
TABLE S6 List of common MQTLs with previous studies.

## Data Availability

All data supporting the findings of this study are available within the paper and within its Supporting Information published online.
