# Supplementary material for: Meta‐QTL s and haplotypes for efficient zinc biofortification of rice
Source: Plant Genome. 2023 Mar 10;16(4):e20315. doi: 10.1002/tpg2.20315 (PMC12807186; doi:10.1002/tpg2.20315)
Supplement: Supplementary file 1 [file TPG2-16-e20315-s001.docx]

**FIGURE S1** Graphical summary of GenFam enrichment analysis

**FIGURE S2** Spatiotemporal expression analysis of selected genes using RiceXPro

**FIGURE S3** Co-expression network of the selected genes

**FIGURE S4** Haplotypes identified for various genes using 3K RG panel

(*OsDMAS1*)

(*OsFRO1*)

(*OsZIP9*)

(*OsMT1g*)

(*OsMT1f*)

**FIGURE S5** Boxplots showing range of zinc content (mg/kg) of various haplotypes across three seasons for the genes used for phenotypic validation. Different alphabet denotes statistically significant differences and vice versa from TukeyHSD test.

**FIGURE S6** Distribution of haplotypes of various genes across different subpopulations in 3K RG panel
